# An electronic medical records study of population obesity prevalence in El Paso, Texas

**DOI:** 10.1186/s12911-022-01781-1

**Published:** 2022-02-22

**Authors:** Jennifer J. Salinas, Jon Sheen, Navkiran Shokar, Justin Wright, Gerardo Vazquez, Ogechika Alozie

**Affiliations:** 1 Department of Molecular and Translational Medicine, Texas Tech Health Sciences Center El Paso, 5001 El Paso Dr., El Paso, TX 79905 USA; 2 Department of Family and Community Medicine, Texas Tech Health Sciences Center El Paso, El Paso, TX USA; 3 Del Sol Medical Center, El Paso, TX USA

**Keywords:** Electronic medical records, Geographic information systems, Obesity, Body Mass Index, Mexican Americans

## Abstract

**Background:**

In this study, we determine the feasibility of using electronic medical record (EMR) data to determine obesity prevalence at the census tract level in El Paso County, Texas, located on the U.S.-Mexico border.

**Methods:**

2012–2018 Body Mass Index (BMI kg/m^2^) data from a large university clinic system in was geocoded and aggregated to a census tract level. After cleaning and removing duplicate EMR and unusable data, 143,524 patient records were successful geocoded. Maps were created to assess representativeness of EMR data across census tracts, within El Paso County. Additionally, maps were created to display the distribution of obesity across the same geography.

**Results:**

EMR data represented all but one El Paso census tract. Representation ranged from 0.7% to 34.9%. Greatest representation were among census tracts in and around clinics. The mean EMR data BMI (kg/m^2^) was 30.1, this is approximately 6% less than the 36.0% estimated for El Paso County using the Behavioral Risk Factor Surveillance Study (BRFSS) estimate. At the census tract level, obesity prevalence ranged from 26.6 to 57.6%. The highest obesity prevalence were in areas that tended to be less affluent, with a higher concentration of immigrants, poverty and Latino ethnic concentration.

**Conclusions:**

EMR data use for obesity surveillance is feasible in El Paso County, Texas, a U.S.-Mexico border community. Findings indicate substantial obesity prevalence variation between census tracts within El Paso County that may be associated with population distributions related to socioeconomics.

## Background

Obesity prevalence has reached an epidemic level across the United States, significantly increasing since the 1990’s [1–3]. This major risk factor for many chronic health conditions, such as diabetes and certain cancers, disproportionately affecting Latino populations [4, 5]. While these trends have been well-documented, little success has been made in mitigating community or individual level risk factors.

Obesity surveillance has been typically conducted using the Center for Disease Control and Prevention (CDC) programs such as the National Health and Nutrition Evaluation Study (NHANES) and the Behavioral Risk Factor Surveillance System (BRFSS) [6, 7]. These and other data sources utilize national cross-sectional health survey methods to collect data on lifestyle behaviors among other health-related metrics. From these data sources, food environment, built environment, segregation, poverty and other contextual risk factors for obesity have been well-established for Latino and other health disparate communities [8–13]. Though this large-scale study design is highly beneficial for national, state, and county level surveillance, it is still limited in providing insight into context of obesity at a smaller geographic scale, i.e. within county or city levels. As place matters in the context of an individual’s health, the more specific the unit of analysis, the better community-based prevention initiatives are able to target high rise areas within cities and counties [14, 15]. Relying on county-level data that is available can make prevention efforts for under-resourced communities futile, since they provide aggregated estimates, not taking into account important variations within a county or city.

Classically, surveillance at a more granular level like census tracts have been conducted in efforts to contain infectious disease spread, such as most recently during the COVID-19 pandemic [16]. GIS and other geographic tracking technologies have been used to track infectious disease within communities across international contexts [16]. These technologies have provided infectious disease researchers and public health departments on-the-ground and real-time information that has guided intervention and prevention programming to curb existing trends within the communities that they serve [17, 18]. In some cases, public health departments have been able to act on real time information and prevent mass exposure to influenza and other viruses. A localized approach such as this applied to the surveillance of obesity would allow for more specific, community-based interventions [18–20]. This may be particularly helpful in areas that have limited resources to conduct wide-scaled intervention efforts.

In 2009, the Health Information Technology for Economic and Clinical Health (HITECH) Act was signed into law as an effort to promote the widespread use of EMR in a “meaningful” way. Aside from the clinical and organizational advantages, EMR enables more available, combined aggregated data across populations leading to better, yet not perfect, health outcome surveillance and research production that benefits overall society [21–25]. Electronic medical records (EMR) is a source for objective data rather than self-reported data, and, could provide a better and low-cost obesity surveillance option for public health departments looking to provide targeted prevention measures.

The purpose of this study is to assess the feasibility and applicability of EMR data obtained from university and county safety-net outpatient electronic medical records to provide census tract-level obesity estimates and distributions across El Paso County, Texas. El Paso, Texas is a unique context in that it is predominantly Mexican American (82%), is socioeconomically disadvantage relative to other cities its size, and has a high prevalence of obesity-related diseases ^26^. This analysis will provide a more detailed picture of the distribution of obesity within the county, facilitating better targeted efforts to reduce obesity.

## Methods

### Design and setting

**Fig. 1:**
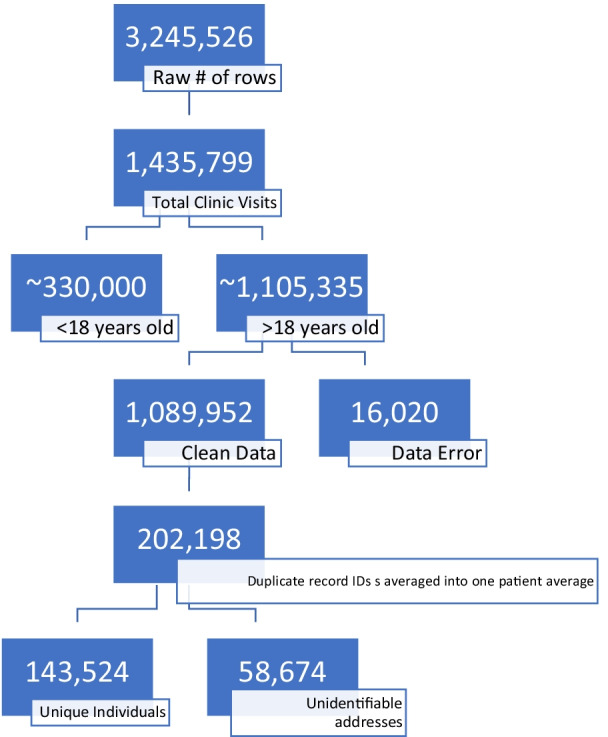
Breakdown of the data cleaning process. 143,524 unique individuals were analyzed from our dataset

2012–2018 adult patient data was extracted from the Electronic Medical Records (EMR) systems from Texas Tech University Health Sciences Center El Paso Clinics and University Medical Center El Paso outpatient clinics in El Paso, Texas. Analysis was completed in 2018–2019. The data process can be seen in Fig. 1. Raw data for over 3.2 million observations were cleaned and prepared for analysis.

### Data preparation

Data cleaning processes are documented in a previous paper [27]. Briefly, duplicates and inconsistencies were either removed or corrected when possible. Any case with unverifiable addresses or incomplete height or weight were removed. Finally, EMR-based Body Mass Indices that seemed out of range (above 100) were recalculated using height and weight or removed completely when data was missing. Patients were assigned census tracts using street addresses. Any unidentifiable addresses were removed. Figure 1 provides and overview of the cleaning process and how we arrived at 143, 524 participants from the 3,245,526 raw case files at the onset of the cleaning process. There were a total of 161 possible census tracts considered for this analysis. Patient representation per census tract was determined by a proportion of patients per census tract by total census tract population. This research was approved by the Texas Tech University Health Sciences Center El Paso Institutional Review Board (IRB) for Human Subjects Research. All methods and procedures were performed in accordance with the IRB guidelines. Since this was a secondary data analysis of existing electronic medical records, requirement for signed informed consent was waived by the Institutional Review Board for Human Subjects Research at Texas Tech University Health Sciences Center El Paso.

### Analysis

Population representation was estimated using total number of patient records divided by total population for each census tract. Average Body Mass Index (BMI (kg/m^2^)) for each of the 161 census tracts was calculated using patient EMR data. Census tract obesity prevalence (BMI (kg/m^2^) 30 or above) was determined for each of the 161 census tracts by dividing the percent of patients with a BMI of 30 or above by the total number of patients represented in each census tract. Census tract patient record representation and obesity prevalence were entered into ArcGIS (ESRI) and mapped for El Paso County.

## Results

**Fig. 2:**
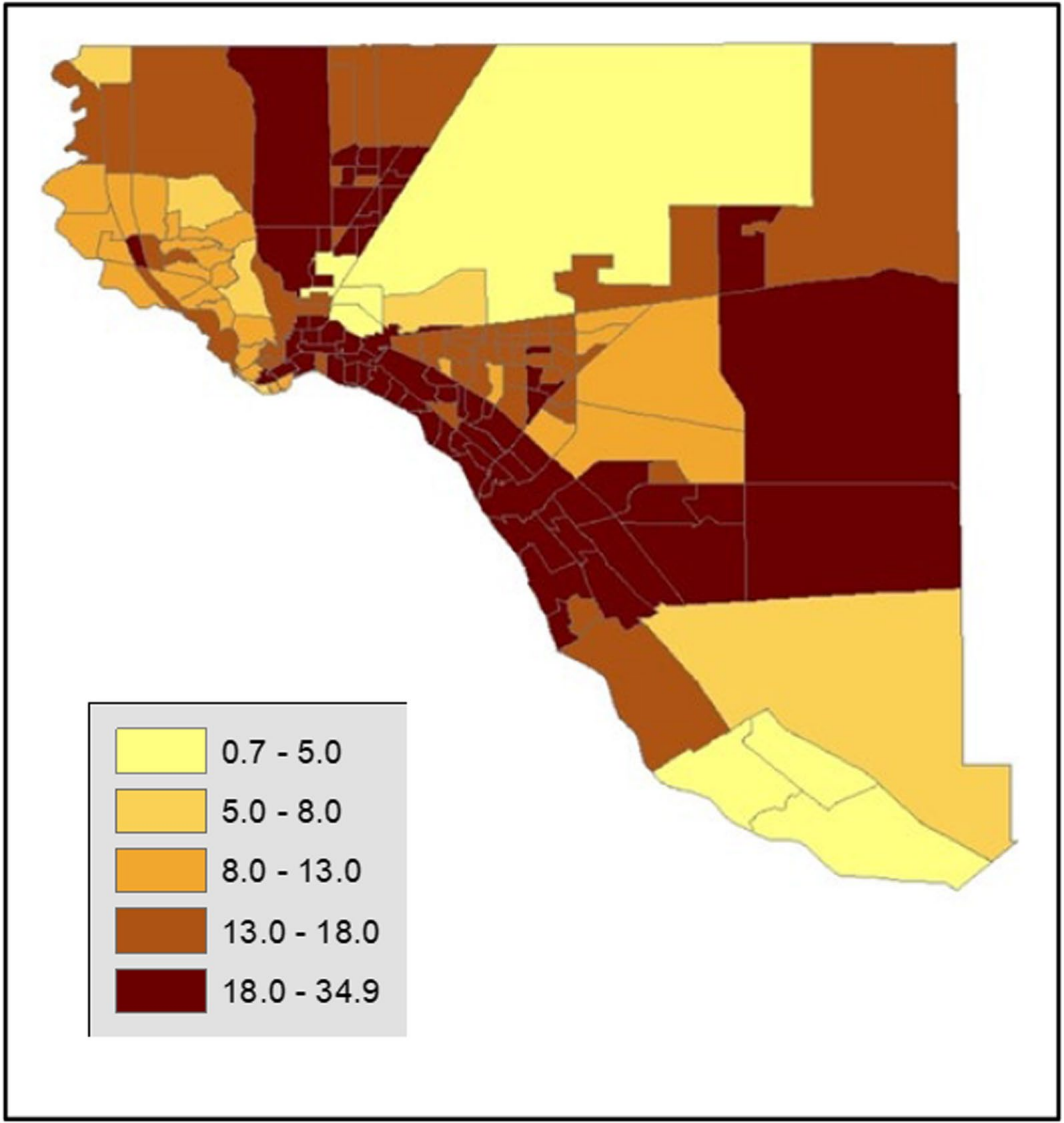
Percent of census tract total represented by EMR patient records

Figure 2 displays the percentage of census tract total population is represented by the EMR patient records. The percent of EMR patients per census tract ranged from 0.7% to as high as 34.9%. Patients were most represented in the Northeast, Downtown/Lower Valley and Far Eastside of El Paso County and in and around clinic catchment areas. Patients were least represented in the Fort Bliss area, the large North-Central area of the map. Fort Bliss generally provides healthcare on base through William Beaumont hospital, rather than community-based clinic. The lack of representation in that area are consistent with what we would expect.

**Fig. 3:**
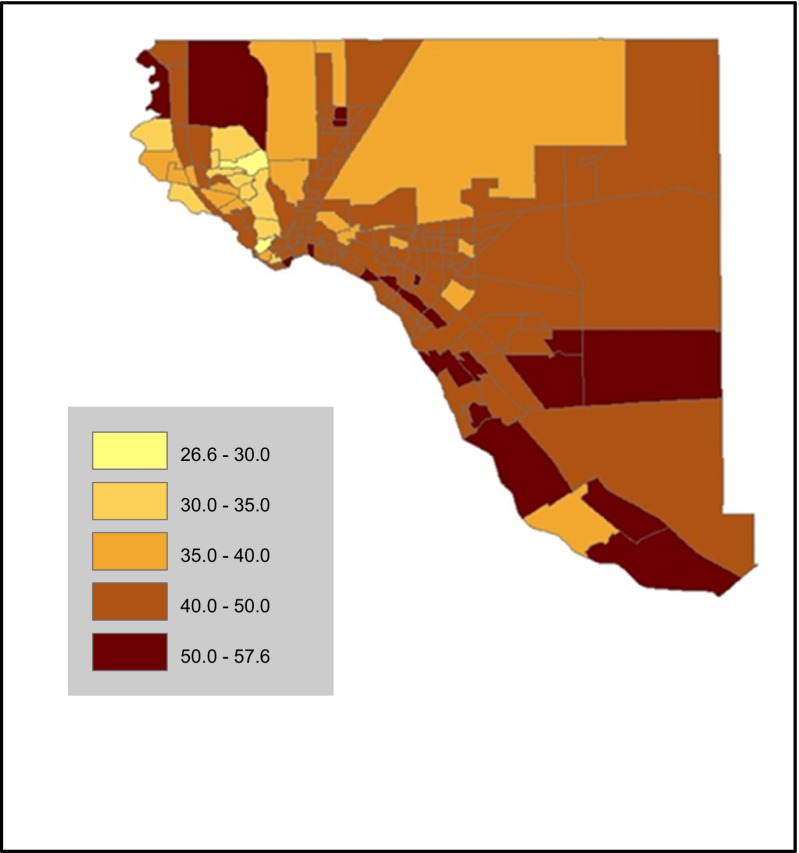
% EMR patient records with 30 or greater BMI (kg/m^2^) by census tract

Figure 3 presents EMR record obesity prevalence by census tract across El Paso County. Prevalence ranged from 26.6% to as high as 57.6%. Census tracts with the highest obesity prevalence were located in the Lower Valley, Far Eastside and Northwest El Paso County. This area corresponds geographically to areas where Latino ethnicity, poverty and immigrant concentration is the highest. On the other hand, the Westside of El Paso County had the pocket with the lowest prevalence, this area is also the most affluent of the County.

## Discussion

Latinos are disproportionately impacted by obesity. Strategies for curbing high prevalence rates among Latinos have generally been informed by data collected at a state or federal level. The continued focus on surveillance at such a high level of aggregation has provided little insight into city or county risk factors that are actionable in addressing current obesity trends. This study provides evidence of feasibility of electronic medical record obesity data as a tool to surveil population-level obesity within small geographic units. Findings from this study also demonstrate the uneven distribution of obesity within small units of a city or county which is not as well represented in national surveillance data at a county level.

Latino communities have been described as obesogenic by multiple previous studies [28, 29]. However, most of these studies have used large-scale sampled data that often only represent urban residing populations or ethnic enclaves within a heterogeneous ethnic structure. This approach has limited the ability to infer factors responsible for obesity in communities where Latinos are the majority. El Paso County Texas is predominantly Mexican American-Latino. While the overall estimated prevalence of obesity is 34.9% based on BRFSS estimates, this study’s findings suggest that there may be substantial variation of the degree of obesity that may co-vary with ethnic concentration and socioeconomic status by census tract (cite other paper). Our study’s findings suggest that known areas of El Paso County that are more heavily concentrated with Latinos, immigrants and lower average socioeconomic status, may also have a higher burden of obesity, relative to the 34.9% overall estimate from the BRFSS. This variation may misrepresent that extent of obesity in areas that may be more heavily populated with Latinos at the same time as being socioeconomically diverse. Our research findings are based on analysis using EMR from a university-base and county outpatient clinic system and may not represent the true county obesity prevalence, since selection in insurance status or presence of chronic conditions may have biased our findings. Therefore, it would be important to replicate this analysis using a pool of EMR from multiple providers. This is an important direct for research given the high burden of obesity in Latino communities and limited effectiveness in curbing trends through currently available interventions.

In this paper, we demonstrated a high feasibility of using a EMR data, to analyze health outcomes across a large population. EMR databases allow for quick extraction and analysis of large quantities of data, and do not require an abundance of resources and manpower, as discussed in Funk et al.’s study [17]. We were able to analyze nearly a fifth of the population -143,524 unique adults in the El Paso area of over 800,000 adults ^26^. While there are shortcomings still with respect to selection bias, this paper’s intent was to demonstrate feasibility, potential use in disease surveillance in areas that otherwise lack this capacity, and provide basis for future surveillance and intervention work.

Using readily available BMI (kg/m^2^) data within university-based and county health clinics allowed us to assess the distribution of obesity within El Paso County. Few studies have previously looked at obesity prevalence with small geographic units such as cities or counties, which is suggested by Jia et al. [14]. Roth et al. [10] successfully explored linking EMR and community data with a large sample size to study factors associated with obesity, but used a zip-code level of analysis. The study by Shafiri et al. [31] is one of the few studies that used both EMR for a large sample and the census tract level for their analysis of the built environment and ethnic disparities in childhood obesity. Funk et al. demonstrated the ease of studying over 380,000 patients from a university-based healthcare system and showed that the results are comparable to NHANES [17]. Our study was an attempt to replicate this approach in a context of disadvantage and high ethnic homogeneity. The previous finds from Shafiri et al., and Funk et al. coupled with the results from the present study demonstrate the feasibility of EMR use in estimating obesity and other chronic diseases at a much more granular level than currently available national estimates at a county level. Future studies are needed to determine the reliability of EMR data to estimate population-level obesity prevalence. The public health implications for this type of use are not only limited to obesity, but to other related health conditions traceable within a patient’s medical record.

This study has a number of strengths. First, the EMR data represented approximately 21% of the overall El Paso population. In some census tracts, the proportion was close to 35%. Secondly, this secondary data source has measured data and not self-reported data like many similar studies. Finally use of existing medical records is a cost effect way to conduct surveillance for obesity and other health outcomes. The findings from this study have a number of limitations that should be noted. First, EMR data were only obtained from an university clinic system clinics and while in many cases the patients represented a large proportion of a given census tract, it may not represent fully the El Paso County population. For example, there was variation in the percentage of census tract population the EMR represented from as low as 0.7% to as high as 35%. It is likely in census tracts where representation was low, there may be substantial variance in the data that may contribute to over or underestimate of obesity. Additionally, patients that visit the clinics on a regular basis, may be sicker and therefore, we may be overestimating the true census tract-level obesity prevalence. This study was intended to demonstrate feasibility in a community that is underrepresented and carries a high burden of chronic diseases related to obesity [29]. Future work using multiple EMR data sources, would reduce potential bias and improve county-wide estimates. Furthermore, analysis in other Latino dominate communities would need to be conducted to determine feasibility and applicability in other settings.

## Conclusions

Use of EMR data for surveillance of obesity prevalence within El Paso County, TX is feasible and may provide a better snapshot of the distribution of obesity within the county than BRFSS estimates. With the potential of using EMR data for obesity and other chronic conditions, public health officials have an opportunity to engage in precision surveillance, identify subpopulations who might be at greatest risk. Including population data such as education, health insurance, mean family income, and other sociodemographic factors could lead to more effective targeted prevention efforts. However, further investigation is needed to determine data quality of these fields in the available EMR databases. This study provides evidence of its potential utility in understanding the distribution of obesity within a Latino community and is a great starting point for further examination of community risk factors for obesity.

## Data Availability

The datasets used and/or analyzed during the current study are available from the corresponding author on reasonable request.
